# Molecular Evolution and Adaptation Strategies in Marine Ciliates: An Inspiration for Cold-Adapted Enzyme Engineering and Drug Binding Analysis

**DOI:** 10.3390/md22110497

**Published:** 2024-11-04

**Authors:** Sandra Pucciarelli, Matteo Mozzicafreddo, Alberto Vassallo, Angela Piersanti, Cristina Miceli

**Affiliations:** 1 School of Biosciences and Veterinary Medicine, University of Camerino, 62032 Camerino, Italy; alberto.vassallo@unicam.it (A.V.); angela.piersanti@unicam.it (A.P.); cristina.miceli@unicam.it (C.M.); 2 Department of Clinical and Molecular Sciences, Marche Polytechnic University, 60126 Ancona, Italy; m.mozzicafreddo@univpm.it; 3 Department of Biology, University of Padova, 35121 Padova, Italy

**Keywords:** marine microorganisms, genomes, cold adaptation, Antarctica, site-directed mutagenesis

## Abstract

In the present review, we summarize genome mining of genomic data obtained from the psychrophilic Antarctic marine ciliate *Euplotes focardii* and its evolutionary-close mesophilic cosmopolitan counterpart *E. crassus*. This analysis highlights adaptation strategies that are unique to the Antarctic ciliate, including antioxidant gene duplication and distinctive substitutions that may play roles in increased drug binding affinity and enzyme reaction rate in cold environments. Enzymes from psychrophiles are usually characterized by high activities and reaction rates at low temperatures compared with their counterparts from mesophiles and thermophiles. As a rule, catalyst cold activity derives from an increased structural flexibility that may lead to protein denaturation in response to temperature fluctuation. Molecular thermolability has been a major drawback of using macromolecules from psychrophiles in industrial applications. Here, we report a case study in which the role of peculiar amino acid substitution in cold adaptation is demonstrated by site-directed mutagenesis. Combined with a rational design approach, these substitutions can be used for site-directed mutagenesis to obtain cold-active catalysts that are structurally stable. Furthermore, molecular docking analysis of β-tubulin isotypes extrapolated from *E. focardii* and *E. crassus* genomes allowed us to obtain additional insight on the taxol binding site and drug affinity. *E. focardii* genome mining and the comparison with the mesophilic sibling counterpart can be used as an inspiration for molecular engineering for medical and industrial applications.

## 1. Introduction

Organisms adapted to inhabit cold environments are named psychrophiles. These organisms thrive despite the challenges posed by freezing temperature, including (i) formation of ice crystals that damage cellular structures, (ii) impaired energy metabolism, and (iii) molecular cold denaturation [1]. The development of genomic technologies has allowed us to gain knowledge on molecular adaptation of microbial communities in the Antarctic ecosystem [2]. The advantage of whole genome sequencing of Antarctic microorganisms lies in the ability to analyze, characterize, and compare the genes in the entire genome using next-generation DNA sequencing methods and bioinformatics tools [2]. Genome mining has also the potential to accelerate new drug discovery [2].

One of the essential adaptive properties of psychrophiles is represented by their cold-active enzymes, which have received significant interest in fundamental research for understanding molecular cold adaptation, responses to environmental stress, as well as their potential applications in industrial processes [1,2]. 

Cold-active enzymes have evolved a range of molecular features that result in heightened structural flexibility, particularly around the active and drug-binding sites. These characteristics lead to low activation enthalpy. In general, cold-active enzymes have lower catalytic maximum temperatures (T_optimum_, 20–45 °C) and higher specific activity at low temperatures. This property can be useful to improve the efficiency of industrial processes by lowering the energetic costs [1,3]. Nevertheless, high structural flexibility may represent a two-edged sword for psychrophilic organisms since it also increases the possibility of protein denaturation in response to temperature fluctuation [4]. Consequently, the pronounced thermolability of cold-active enzymes has been a major drawback for their applications in industrial contexts [5]. To solve this issue, a viable solution may involve the modification of existing cold-active enzymes by site-directed mutagenesis combined with a rational design approach, aimed to achieve intended objectives [6]. 

Several techniques have been reported to perform rational engineering, including site-directed mutagenesis, which is a powerful technology used for modifying proteins to make them more suitable for peculiar applications or purposes than their unmodified counterparts [7]. While these biotechnologies are all well assessed, the challenge lies in selecting residue mutations and predicting the impact of individual substitutions on protein properties and functions. Achieving the desired result may require measuring the effects of several substitutions, which usually are not readily attained. The study of genome molecular evolution in Antarctic psychrophilic microorganisms contributes to the rapid identification of substitutions that enable proteins to function at freezing temperatures. The identified substitutions can be used to engineer enzymes and achieve the desired outcomes in industrial applications.

Genome mining of Antarctic microorganisms can be useful also to study drug-binding kinetics of cold-adapted molecules. For example, microtubule polymers (the main component of the eukaryotic cell cytoskeleton) bind several drugs that control their polymerization/depolymerization dynamic. Taxol, a drug extracted from the ground bark of *Taxus brevifolia*, is a stabilizing microtubule agent that blocks cell mitosis by binding to the β-tubulin subunit of the heterodimer, making it a powerful chemotherapy agent. However, the polymerization/depolymerization dynamic of microtubules from psychrophilic organisms is completely different from that of homeothermic animals (organisms that live at temperate environments): cold-adapted microtubules are able to polymerize from the α- and β-tubulin heterodimers and remain stable at low temperatures, whereas the assembly of microtubules from homeothermic organisms requires physiological temperatures (30–37 °C), and those microtubules usually disassemble at temperatures below 4 °C. Therefore, these different microtubule dynamic properties may interfere with drug-binding affinity.

Antarctica provides a unique natural laboratory to investigate the evolutionary processes behind environmental adaptation [8]. The fragmentation of Gondwana and the development of wide-scale glaciation from the warm Late Cretaceous (100.5–66 Ma) have resulted in major episodes of extinction and adaptation to the present polar environment [9]. The isolation of the Antarctic continent after the establishment of the Antarctic Circumpolar Current, which acts as a barrier, led to the evolution of endemic marine and terrestrial biotas without exchange with the rest of the planet [9]. Consequently, Antarctic marine organisms have adapted to cope with both glaciation and oxidative stress. The latter is a particularly severe threat to marine organisms inhabiting Antarctica because they are exposed to high dissolved oxygen and to intense UV radiation [10,11].

In this review, we summarize the outcomes derived from the genome sequence comparisons and mining between the psychrophilic Antarctic ciliate *Euplotes focardii* and the mesophilic congeneric evolutionary-close species *E. crassus*. These two organisms are the optimal system for this study: they are closely related eukaryotic microorganisms, but they are adapted to different environments. The comparison reveals specific characteristics, gene duplication, and amino acid substitutions associated with cold adaptation, oxidative stress strategy, and molecular thermal stability. Furthermore, our analysis allowed us to identify differences in drug interaction. These findings not only provide insights into the molecular adaptations of these organisms to different temperatures but also offer potential targets for site-directed mutagenesis that can be employed to optimize the function of industrially relevant enzymes at various temperatures and stress conditions. Furthermore, this comparison provides insight on drugs binding at different temperatures. Understanding these sequence variations paves the way for the development of more robust and versatile enzymes tailored to specific industrial requirements and drug development.

## 2. The Importance of Marine Ciliates: Antarctic and Mesophilic *Euplotes* Species

Ciliates represent a group of eukaryotic microorganisms widespread in nature. Large ciliate communities are also present in the marine Antarctic environment [12,13]. Differently from yeast, ciliates are considered the most complex unicellular eukaryotes for their cell architecture and molecular systems involved in processes such as ciliary beating, digestion, excretion, and sexual phenomena. Being directly exposed to environmental cues, ciliates provide excellent material to study adaptive evolution to cold. The most deeply studied cold-adapted ciliate is the Antarctic marine *Euplotes focardii*, originally isolated from the coastal seawaters of Terra Nova Bay and classified as an obligate psychrophilic stenothermal. It survives and reproduces optimally at 4–5 °C and has a genome rich in A/T base pairs [14]. Furthermore, the sexual phenomenon of conjugation shows traits rather unusual for *Euplotes* species. In *E. focardii*, only one of the two pair members was shown to carry out fertilization and to give rise to a new clone of vegetative cells. The other underwent cell body shrinking after 4–5 days of union, lost the locomotory ciliary apparatus, and eventually died [15]. *E. focardii*, as well as all hypotrich ciliates, possess two nuclei: (i) the germline micronucleus (MIC), containing the entire genome in the form of large chromosomes, and (ii) the somatic (active) macronucleus (MAC), containing small linear DNA molecules, most of which constitute a single genetic unit flanked by regulatory regions and capped by telomeres [14]. Thus, the genomes of *E. focardii* and congeneric species are enriched in genomic units, each yielding thousands of homologous protein-coding and gene-regulatory sequences, which can be systematically mined for molecular adaptations to cold temperatures. The availability of the genome of *E. crassus* [16], a mesophilic species that is very close from an evolutionary viewpoint, provides a unique opportunity for protein sequence comparison.

Research on *E. focardii* has provided molecular insights on microtubule stability at low temperature, on constraints exerted by the cold to the process of tubulin protein folding, and on responses to the heat and oxidative stresses (see description and references in Table 1). Moreover, *E. focardii* can be studied also for mining the molecular basis of enzyme cold adaptation, as reported in the paragraphs below.

### Genomes from Euplotes: Insight into Gene Duplication Events, Adaptation, and Stress Response

The MAC genome from the Antarctic psychrophile *E. focardii* and the mesophilic congeneric species *E crassus* have been sequenced [14]. The two genomes were compared to characterize differences in gene content that may be consequent to cold adaptation and defense to stress. This analysis mainly focused on tubulin, antioxidant enzymes, and heat shock protein (HSP) 70 gene families. It resulted that the α-tubulin genes and those encoding SOD and CAT antioxidant enzymes are more numerous in the *E. focardii* genome than in the mesophilic *Euplotes* species. For what concerns SOD and CAT antioxidant enzymes, we also expanded our comparison with two additional mesophilic *Euplotes* species, *E. vannus* and *E. octocarinatus* (Table 2). As reported in Table 2, the number of antioxidant enzymes is much higher in *E. focardii* than in other mesophilic *Euplotes* species. As a matter of fact, Antarctic marine microorganisms must cope with oxidative stress due to the higher solubility of dissolved oxygen in cold seawaters [14]. Therefore, Antarctic microorganisms must have evolved molecular mechanisms to neutralize reactive oxygen species. The *E. focardii* SOD and CAT encoding gene families appeared composed by a higher number of genes with respect to the mesophilic *E. crassus*, probably due to a repeated event of gene duplication. 

**Figure 1:**
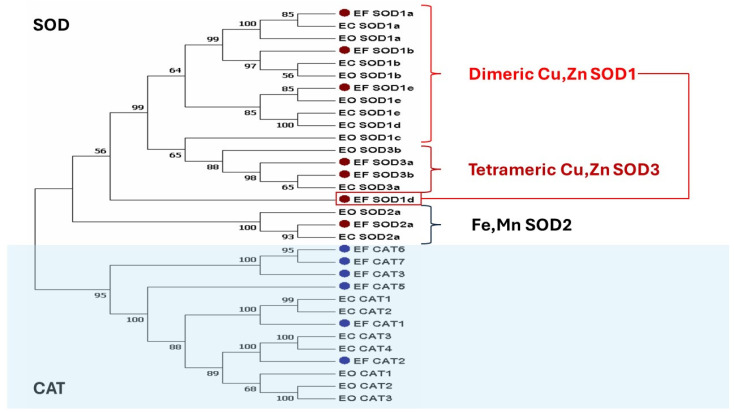
Phylogenetic analysis of *E. focardii* SODs (red dots) and CATs (blue dots) compared with homologous sequences from *E. crassus* and *E. octocarinatus*. SODs are grouped into three protein families, based on the metal cofactor they contain and on the protein folding. Copper, zinc SODs (Cu,Zn SODs) are found in the cytoplasm of eukaryotes, in the chloroplasts of some plants, and in the periplasmic space of bacteria [11]. This group of SODs is often referred to as SOD1. SOD1s are homodimers. SOD3s, the Cu,Zn enzymes present in the extracellular fluids of eukaryotes, are similar to the previous ones but have tetrameric quaternary structure. Iron- and manganese-containing SODs (FeSOD and MnSOD) are referred to as SOD2 [11].

Among the SOD genes, the SOD3 family appears the most “amplified” (Figure 1). SOD3 family members are found in the extracellular matrix and prevent cell damage by extracellularly reactive oxygen species (ROS). However, the higher number of gene duplication and differentiation is clearly visible for the genes encoding catalases (CATs) (blue dots in Figure 1), the major cell defense against hydrogen peroxide. In *E. focardii*, the CAT family is encoded by six genes versus four and three in *E. crassus* and *E. octocarinatus*, respectively. Furthermore, four of the SOD genes form a separated clade in the phylogenetic tree, indicating a high degree of sequence differentiation. 

On the other hand, the *hsp70* genes in *E. focardii* are fewer in number and not inducible by thermal stress but only to oxidative stress compared with mesophilic *Euplotes* [14]. All these results suggest that molecular adaptation to cold and oxidative stress in the Antarctic environment may be due to not only peculiar amino acid substitutions (see next chapters) but also duplication and divergence of paralogous genes. Furthermore, Kim et al. suggest potential roles for paralogy in environmental adaptation: genomic expansions of specific protein gene families may contribute to physiological fitness in freezing polar conditions, as previously reported for Antarctic notothenioids [21].

## 3. Enzyme Sequences

### 3.1. In Silico Comparative Analysis on Euplotes Hydrolytic Enzymes

Hydrolytic enzymes are one of the most investigated groups of catalysts because they display both hydrolysis and synthesis activity toward many useful ester compounds [22]. These enzymes catalyze esterification, trans-esterification, interesterification, acidolysis, alcoholysis, and aminolysis, in addition to the hydrolytic activity on triglycerides [22]. Therefore, they have emerged as key enzymes for a wide variety of applications, including synthesis of biopolymers and biodiesel, production of pharmaceuticals and detergents, food processing, chemical transformation, and cosmetics. Hydrolytic enzymes from cold-adapted organisms have attracted attention for industrial applications to produce compounds that are frail at high temperatures [23]. Furthermore, certain hydrolytic enzymes are particularly relevant for industrial applications since they can be stable in organic solvents such as methanol, ethanol, DMSO, and n-hexane [24,25].

Hydrolytic enzymes have also been used as a benchmark for examining the structural basis of cold adaptation [26]. The comparison of amino acid sequences between homologous enzymes from psychrophilic and mesophilic organisms often reveals which structural changes make the molecule suitable to work efficiently in the cold. However, when the comparison is performed with a few enzymes, it may provide contrasting results about the role of local structural flexibility and amino acid substitutions. 

An in silico analysis that includes a high number of homologous sequences may help in this contest: 46 lipases from *E. focardii* genomic sequences were compared with 58 lipase sequences from the *E. crassus* genome [16]. These lipases were: (i) patatin-like phospholipases; (ii) α/β-hydrolase associated lipases, and (iii) esterases (Table 3) [27]. Out of the 46 lipases from *E. focardii*, 9 were determined to be patatin-like phospholipases, 29 αβ-hydrolase associated lipases, and 8 esterase lipases. Out of the 58 lipases from *E. crassus*, 17 were identified as patatin-like phospholipases, 28 αβ-hydrolase associated lipases, and 13 esterase lipases (summarized in Table 3). 

**Figure 2:**
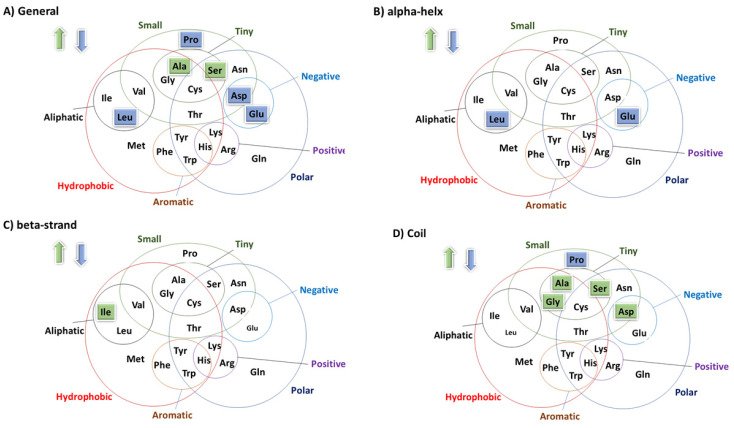
Amino acid substitution trend in *E. focardii* hydrolytic enzymes, obtained from Tables S1 and S2. (**A**) General substitution trend; (**B**,**C**) substitution trends in the alpha-helix and the beta-strand secondary structures, respectively. (**D**) substitution trend in the coil regions. The residues that in *E. focardii* lipases are in higher or lower number with respect to the homologs from *E. crassus* are highlighted in green and cyan, respectively.

The amino acid composition, as well as the predicted secondary and tertiary structures of these lipases, was compared to extract relevant information related to cold adaptation (Figure 2). The applied in silico approach is described in detail in the supplementary materials. The results of this analysis confirmed the preference for small amino acids such as Ala and Ser and the reduced presence of Pro residues in the primary structure of *E. focardii* enzymes. Furthermore, it enabled us to identify some new factors correlated with the secondary structure possibly responsible for enhanced enzyme activity at low temperatures. 

In the α-helices of *E. focardii* lipases, the amino acids Glu and Leu show significantly low frequencies (Figure 1). Glu and Leu residues favor and stabilize the formation of helical structures [28], and, consequently, these residues tend to decrease molecular flexibility. Charged amino acid group residues, such as Glu, are known to contribute to ion pair electrostatic interactions and maintain conformation stability in protein surfaces [28] that limits structural flexibility in the cold. A reduced number of Glu and Asp have been reported also for *Halorubrum lacusprofundi*, an Archeon isolated from the Deep Lake in Antarctica [29]. *E. focardii* lipase beta-sheets did not show any significant changes as compared with *E. crassus*, except for an increase in frequency of the amino acid Ile (Figure 2). Increased exposure of hydrophobic residues to the solvent enhances protein solvation, which is considered a characteristic of cold-adapted enzymes [28]. In organisms living in cold, environment, the role of several previously recognized amino acid substitutions for cold adaptation, specifically Gly, Ala, and Ser, and the reduction in Pro residues has been confirmed. 

In summary, the in silico comparison allowed the identification of residues that are favored in enzymes from psychrophiles, proposing Leu (decreasing) and Ile (increasing) as new protagonists. However, biochemical assays are necessary to verify their effective role in cold adaptation. Studies performed on amylases and lipases from *E. focardii* and *E. crassus* allowed the biochemical analysis of their importance in cold activity and thermostability [30]. The approach used was to modify *E. focardii* cold-adapted enzymes by site-directed mutagenesis combined with a rational design approach [31], which generally is based on structure-guided consensus sequence alignments with sequences that have moderate or high amino acid identity, to reduce the number of possible target residues to be mutated.

### 3.2. Importance of Distinctive Substitutions

#### 3.2.1. The Role of Pro and Ile Residues in the Patatin-like Phospholipase Thermal Activity and Stability

**Figure 3:**
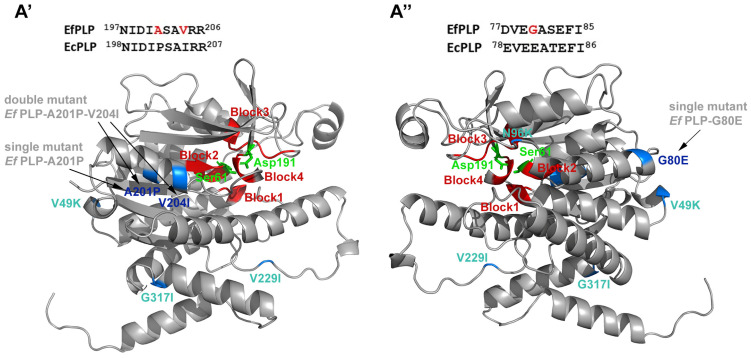
Low-resolution homology model of EfPLP shown from the two opposite views ((**A’**,**A’’**) represent the right and the left side of the lipase structure, respectively. Modified from [30]). Side chains of the catalytic dyad aspartate and serine are shown in green; the conserved patatin block domains (Block I, Block II, Block III, and Block IV) are shown in red; the mutated residues are shown in blue in the 3D structure and in red in the partial sequence alignment. Other substitutions of amino acids that may influence intramolecular flexibility are shown in light blue. The methods used for the in silico analysis are reported in the original papers.

The patatin-like phospholipases from *E. focardii* (EfPLP) and *E. crassus* (EcPLP) possess both phospholipase and esterase activities in a wide range of pH conditions making them good candidates for the implementation of new and harsh industrial applications [30]. The two proteins are 80.67% identical, and both EfPLP and EcPLP possess conserved blocks (indicated as blocks I, II, III, and IV in Figure 3). Block I consists of a Gly-rich region, which probably serves as an oxyanion hole. Block II represents a typical lipase nucleophilic elbow Gly-X-Ser-X-Gly containing the putative active-site Ser (Gly59-Val60-Ser61-Ala62-Gly63 in EfPLP and Gly60-Val61-Ser62-Ala63-Gly64 in EcPLP). Block III contains a conserved ASXXXP motif of unknown function. Block IV contains the active-site Asp (Asp191 in EfPLP and Asp192 in EcPLP) that, together with the active-site Ser, forms the catalytic Ser-Asp dyad.

Yang et al. [30] tested the engineered versions of EfPLP in which the unique residues of EfPLP, Gly80, Ala201, and Val204 were substituted through site-directed mutagenesis with residues found in the *E. crassus* homolog (Glu, Pro, and Ile, respectively). Site-directed mutagenesis was performed by PCR using primers designed with the corresponding mutations. The protocols are reported in the Material and Methods of the original papers [30]. Additionally, three corresponding mutants of EcPLP were also generated and characterized, two single and one double mutant (Figure 3). 

As summarized in Table 3, the substitution of EfPLP small amino acids Ala and Val with the rigid Pro and the bulky residue Ile impaired cold activity: consequently, the optimal temperatures increased above 30 °C. In addition, these changes enhanced the thermostability since the half-life of the enzymes at 70 °C was above that of the wild type. The opposite effects were recorded for EcPLP by the introduction of the residues of EfPLP: indeed, the enzyme acquired cold activity at 0 °C, but its half-life decreased. Furthermore, EfPLP and EcPLP show the characteristics of psychrophilic and mesophilic phospholipase, respectively, since the EfPLP optimal temperature activity is 25 °C, whereas that of EcPLP is 35 °C (Table 4).

This work confirmed the role of small amino acids on molecular cold adaptation. Furthermore, this study contributed to the understanding of the ambiguous role of Ile in EfPLP: it may represent a key substitution to find a balance between cold activity and thermostability, since the replacement of Val with Ile maintained an efficient catalysis in the cold, with an increased half-life at 70 °C.

#### 3.2.2. The Role of Pro and Thr Residues in the *E. focardii* α-Amylases

α-Amylases can be obtained from several sources, including plants, animals, and microorganisms. The protein is described as having 3 domains: A, B, and C. A is a (β/α) 8-barrel; B is a loop between the β3 strand and the α3 helix of A; C is the C-terminal extension characterized by a Greek key. Most of the enzymes have an active site cleft found between domains A and B where a triad of catalytic residues (Asp, Glu, and Asp) performs catalysis.

Microbial α-amylases are generally attractive for biotechnological and industrial applications [32,33], particularly those produced by extremophiles, as they can withstand harsh conditions [34]. The α-amylases have been utilized in a wide range of industrial processes, such as food, detergent, textile, and paper industries [35]. They represent approximately 30% of the world’s enzyme market [36].

**Figure 4:**
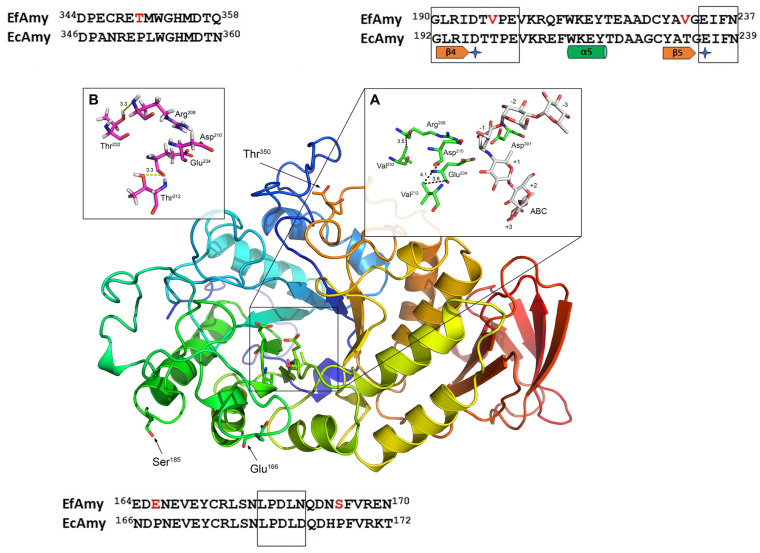
Low-resolution homology model of EfAmy (modified from [37]). EfAmy residues that were chosen for mutation into Pro are indicated in black in the 3D structure and in red in the partial sequence alignment. Alignment of the predicted amino acid sequences of EfAmy and EcAmy conserved regions is boxed. Secondary structure elements are presented below the sequences. (**A**) The catalytic dyad (Glu234 and Asp210) is indicated by the blue cross. (**B**) The same region shown in panel A with V212T and V232T mutations. The methods used for the in silico analysis are reported in the original papers.

The α-amylases from *E. focardii* (EfAmy) and *E. crassus* (EcAmy) are 66.67% identical. The conservation between EfAmy and EcAmy is not uniform throughout the entire sequence. Seven regions of conservation have been identified, two of which are boxed in Figure 4.

EfAmy and EcAmy show the characteristics of psychrophilic and mesophilic phospholipases, respectively: the EfAmy optimal temperature activity is 25 °C, whereas that of EcAmy is 35 °C ([37] and Table 4). Yang et al. [37] also tested engineered versions of the α-amylases. In this case, the role of Pro and Thr residues was checked. The substitutions of the small amino acid Val in the EfAmy sequence with Pro and Thr remarkably impaired cold activity only when these mutations were combined (Table 5). This study confirmed that Pro rigidifies molecular structure, conferring thermostability since the half-life almost doubled in the mutant containing three additional Pro. Furthermore, it proposed Thr residue as a new potential substitution for combining cold activity and thermostability.

#### 3.2.3. The Ambiguous Role of Ile in Protein Cold Adaptation

As summarized in Table 4, the substitution of EfPLP small amino acids Ala and Val with the bulky residue Ile impaired the enzyme cold activity. At the same time, the in silico analysis that includes a high number of homologous sequences of hydrolytic enzymes performed by Yang and co-workers revealed an increase in the frequency of the amino acid Ile (summarized in Figure 2 and Figure S1, and Table S1) [27]. Being hydrophobic, Ile residues are usually buried in protein hydrophobic cores. Furthermore, Ile is a β carbon branched amino acid; that means that it contains two non-hydrogen lateral groups attached to the β carbon of the amino acid, as Val and Thr. The most pronounced effect of this is that the Leu side chain is rigid and, therefore, is preferentially found within β-sheets. The Ile side chain is very non-reactive, and, thus, it is rarely directly involved in protein function, though it can play a structural role by modifying protein conformation.

How can we explain the relation between increased Ile frequency in hydrolytic enzymes from an Antarctic strictly psychrophile and cold adaptation? As reported above, molecular cold adaptation can be ascribed to peculiar amino acid composition, i.e., reduced content of Pro and Arg residues, and a higher number of small amino acids. However, it can be consequent to a decrease in the hydrophobic core compactness, an increase in the number of solvent-exposed hydrophobic side chains, and longer and more hydrophilic loops (summarized in [38]. In other words, Ile residues may confer conformational changes at the tertiary and quaternary structures of enzymes from psychrophiles, allowing them to be functional at low temperatures [38].

**Figure 5:**
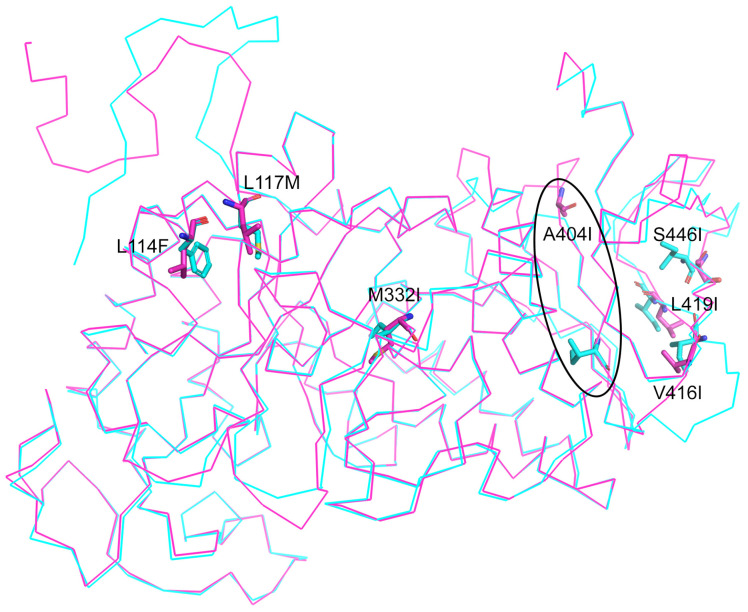
Superimposed low-resolution homology model of EfAmy (in light blue ribbon) and EcAmy (in violet ribbon). The circle highlights the A404I substitution (where A is the EcAmy residue, and I is the EfAmy substitution). The methods used for the in silico analysis are reported in the original papers.

Figure 5 shows the superimposition of EfAmy and EcAmy three-dimensional structure: high structural divergence is clearly visible at the level of the substitution A404I (where A is the EcAmy residue, and I is the EfAmy substitution). Also, V416I and L419I substitutions contribute to structure conformation changes, even though at low extent. The role of Ile in cold adaptation has never been reported before. The only report is about the importance of isoleucine for *Bacillus subtilis* to survive cold shock from 37 to 15 °C by modifying the cell membrane structure [39]. Furthermore, α-amylases have been only studied from cold-adapted bacteria [40]. Therefore, this is one of the few reports of a possible role of Ile residues in molecular structural changes and cold adaptation.

## 4. Future Research Directions: Insights on the Relation Between Cold-Adapted Substitutions and Drug-Binding Affinity from the β-Tubulin Taxol Binding Site

The distinctive amino acid substitutions observed in *E. focardii* polypeptides are thought to result from adaptive molecular evolution, enabling *E. focardii* enzymes to efficiently function at low temperatures. A key question, particularly relevant to the potential application of these cold-adapted molecules, is whether these substitutions also impact their binding affinity for drugs. To explore this, we investigated the β-tubulin binding affinity for taxol, a chemotherapeutic drug that targets microtubules. 

**Figure 6:**
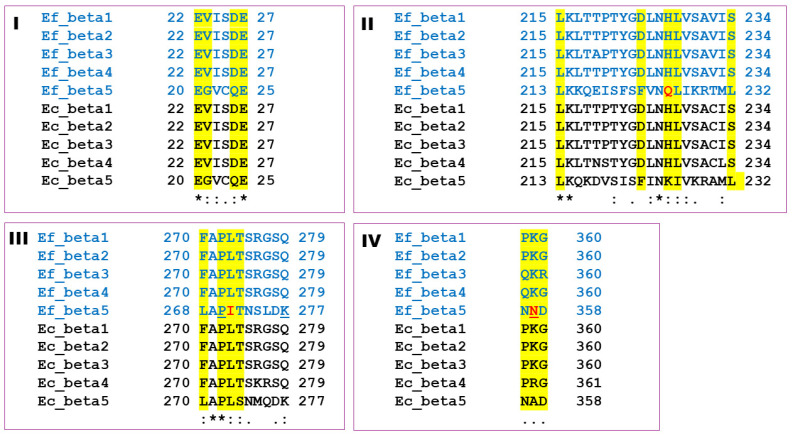
Taxol binding domains (**I**–**IV**) sequence alignment of the *E. focardii* (cyan) and *E. crassus* (black) *β*-tubulin isotypes. Highlighted in yellow are the residues that form the taxol binding site. In red are the unique residues in *E. focardii* Beta5. The *E. focardii* Beta5 residues that are involved in the interaction with taxol as reported in Figure 6 are underlined. Alignment done using Clustal Omega (modified from [43]).

Taxol is a microtubule stabilizing drug, and it is an important chemical biological tool for the chemotherapy of breast, lung, and ovarian cancer [41]. The taxol binding site on β-tubulin was resolved by cryo-electron microscopy by Nogales and collaborators [42], and this finding allowed us to identify the residues directly involved in the drug binding to *E. focardii* and *E. crassus* β-tubulin isotypes. By genome mining, five β-tubulin isotypes were identified in both *E. focardii* and *E. crassus* [14]. The comparison of residues involved in taxol binding is reported in Figure 6. From this analysis, the beta isotype 5 (Beta5) binding domains from both *Euplotes* species appear as the most divergent in amino acid composition (Figure 6). 

It has been reported that taxol acts differently on different human tubulin isotypes [41]. The differing responses of distinct isotypes to taxol show that a small set of conservative residue substitutions change the apparent affinity of microtubules for taxol [41]. Considering the presence of unique substitutions in *E. focardii* and *E. crassus* Beta5, we estimated the relative binding affinity for taxol by docking analysis using Autodock Vina, NNScore2, and SwissDock algorithms. In general, the binding affinity of the *E. focardii* beta tubulin isotypes resulted weaker with respect to that from *E. crassus*, except for the Beta5 that binds taxol with higher affinity (indicated by the low concentration of taxol reported in Table 6). Conversely, the *E. crassus* Beta5 has a very low binding affinity for this drug, besides the similarity with the *E. focardii* Beta5 in residue composition, suggesting that the *E. focardii* unique substitutions may be responsible for this different behavior.

**Figure 7:**
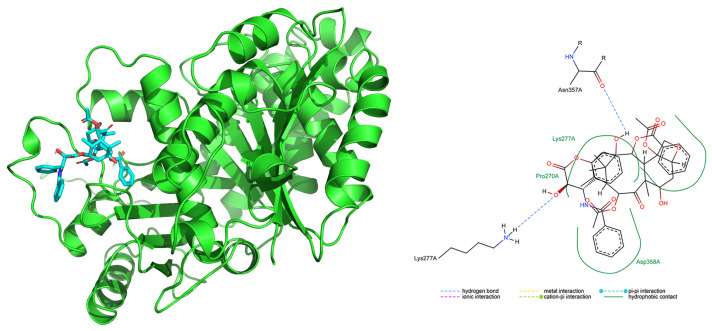
3D and 2D representations of the complex between taxol and *E. focardii* beta tubulin isotype 5. Taxol is represented in cyan in the lateral view of the beta tubulin 3D structure. Interaction types of the involved residues are described under the 2D scheme. The methods used for the in silico analysis are reported in the original papers.

Figure 7 represents the 3D model of the complex between taxol and *E. focardii* beta tubulin isotype 5, where taxol is represented in cyan. The taxol binding site is close to the M-loop that is involved in lateral interaction among protofilaments to form microtubules [42]. Therefore, the taxol binding site in this position can stabilize lateral connections between protofilaments [41]. The 2D representation of the docking analysis (Figure 7) reveals that, among the unique substitutions in *E. focardii* beta tubulin isotype 5 reported in red in Figure 6, Asn357 is directly involved in the interaction with taxol, supporting its possible role in the strong taxol binding affinity of this isotype.

## 5. Conclusions

This review underscores the significance of genome mining of closely related species adapted to different marine environmental conditions to detect gene duplication events, minimal sequence changes, and structural modifications at the base of molecular cold adaptation. All the differences and mutation sites identified were selected based on structural and consensus analyses of the cold-adapted molecules and their mesophilic counterparts. The knowledge obtained from this analysis may have a large plethora of applications. For example, the rational design of enzymes requires a thorough understanding of molecular evolution to identify mutations that will lead to desired enzyme properties, particularly the elevated thermostability and catalytic efficiency, both crucial for industrial applications. Unlike the classic site-directed mutagenesis, which relies solely on protein structural analysis that often yields adverse impacts on kinetic parameters, the structure-guided consensus approach assumes that nature has efficiently optimized the protein’s sequence space, thus making adapted proteins suitable for industrial applications through protein engineering strategies, frequently employed to optimize enzyme traits. 

This study emphasizes the importance of delving into molecular evolution and adaptation to better identify the residues that may work as targets of enzyme engineering. Furthermore, it provides some clues that may help in the understanding of molecular mechanisms underlying the difference in drug-binding affinity. We reported the specific case of the taxol binding site on microtubules, revealing possible residue substitutions in the two *Euplotes* species under study that can be responsible for a different binding affinity of the drug. The information provided in this review may also contribute to the understanding of the basic molecular mechanisms of drug resistance. This is particularly important in the specific case of taxol to explain the evidence of taxol resistance related to microtubule differences, a major obstacle to improving the overall response and survival of cancer patients.

## Figures and Tables

**Table 1:** *Euplotes focardii* unique cold adaptation features.

Feature	Description	References
Specific localization and roles of β-tubulin isotypes in microtubules	Low temperatures impair microtubule assembly from the αβ-tubulin dimer subunits. In *E. focardii,* conserved β-tubulin isotypes are involved in the formation of all microtubular structures, i.e., cytoskeleton, spindles, and cilia. By contrast, a unique β-tubulin isotype, named EFBT3, specifically localizes at the bases of cilia promoting microtubule polymerization.	[17]
Non-canonical β-tubulin folding	Low temperatures exert physicochemical constraints on the process of protein folding. To attain its native monomeric structure, β-tubulin needs the assistance of the eukaryotic class II chaperonin CCT and the cofactor A (CofA). The rare Cys281 of the unique EFBT3 isotype is critical for its correct folding. Furthermore, additional factors are required for the completion of the process, besides CCT and Cofactor A.	[18]
Cold-active enzymes are thermotolerant and even thermostable	Cold-active enzymes are often heat-sensitive and undergo inactivation and unfolding even at mild temperature. In *E. focardii*, superoxide dismutases (SOD), ubiquitous metalloenzymes that rapidly respond to oxidative stress, combine cold activity, local molecular flexibility, and thermotolerance to face oxidative stress, since these enzymes are active at 4 °C, and display melting temperatures and residual enzyme activity in the temperature range of 50–75 °C.	[11]
Evolutionary loss of heat shock proteins 70 (Hsp70) gene induction by heat stress	*E. focardii* is an extreme stenotherm that has lived for a long period in a stable, chronically cold environment. This evolutionary history probably caused the loss of the capacity to induce *hsp70* mRNA accumulation and a corresponding increase in Hsp70 protein production in response to temperatures above its physiological norm. However, the *E. focardii hsp70* gene remains inducible by several chemical stressors, including sodium arsenite, tributyltin, and hydrogen peroxide.	[19,20]

**Table 2:** Number of antioxidant enzymes predicted in ciliate organisms phylogenetically close to *E. focardii*. Modified from [14]. SOD: superoxide dismutase; CAT: catalase; PRX: peroxiredoxin; TRXR: thioredoxin; GR: glutathione reductase: GPx: glutathione peroxidase; GS: glutathione synthetase; GST: glutathione S-transferase; TGR: thioredoxin–glutathione reductase.

	SOD	CAT	PRX	TRXR	GR	GPx	GS	GST	TGR	Total
*Euplotes focardii*	**7**	**6**	5	2	2	5	3	**69**	0	**99**
*Euplotes crassus*	6	4	6	3	2	7	3	60	0	91
*Euplotes vannus*	6	5	4	2	2	3	0	31	0	53
*Euplotes octocarinatus*	6	3	4	9	0	4	1	63	3	93

**Table 3:** *Euplotes focardii* hydrolytic enzymes used for the in silico comparative analysis.

Enzyme Family	Characteristics	Function	Number of Enzymes	
			*E. focardii*	*E. crassus*
Patatin-like phospholipases (PLPs, EC = 3.1.1.3, PFAM accession number: PF01734)	Molecular weight: ~40 kDa. Mean sequence length: 358.4 AA. PDB structure 1OXW	The PLPs are nonspecific lipid acyl hydrolases predominately found in potato tubers where they are essentially storage proteins with catalytic properties. These proteins catalyze the nonspecific hydrolysis of phospholipids, glycolipids, sulfolipids, and mono- and diacylglycerols.	9	17
α/β-hydrolase associated lipases (PFAM accession number: PF00561)	Molecular weight: ~40 kDa. Mean sequence length: 392.3 AA PDB structure 1K8Q	Enzymes with a broad variety of catalytic activities such as acetylcholinesterases, acyltransferases, amidases, dehalogenases, dienelactone hydrolases, epoxide hydrolases (EH), esterases, hydroxynitrile lyases, lipases, peroxidases, proteases, and thioesterases.	29	28
Esterase lipases (carboxyl ester hydrolases, EC 3.1.1.1)	Molecular wight: ~40 kDa. Mean sequence length: 394.5 AA PDB structure 6A0W	Esterase catalyzes the hydrolysis and synthesis of short-chain and partly soluble aliphatic esters.	8	13

**Table 4:** Effect of temperature on the esterase activity of *E. focardii* (EfPLP) and *E. crassus* (EcPLP) wild-type and mutant patatin-like phospholipases. Each mutation is indicated by the amino acid in the wild type followed by its position in the primary structure and by the mutated amino acid (data were extrapolated from [30]).

	Relative Activity (%)						Half-Life
**EfPLP**	**0 °C**	**25 °C**	**30 °C**	**35 °C**	**38 °C**	**50 °C**	**Hours at 70 °C**
Wild type	50%	100%				0%	2.07
EfPLP G80E	48%	100%				35%	2.21
EfPLP A201P	48%		100%			50%	2.19
EfPLP A201P/V204I	55%			100%		60%	2.31
**EcPLP**	**0 °C**	**25 °C**	**30 °C**	**35 °C**	**38 °C**	**50 °C**	**Hours at 70 °C**
Wild type	0%				100%	50%	2.57
EcPLP E81G	0%			100%		30%	2.45
EcPLP P202A	0%		100%			25%	2.48
EcPLP P202A/I205V	22%	100%				18%	2.28

**Table 5:** Effect of temperature on wild-type α-amylases from *E. focardii* (EfAmy) and *E. crassus* (EcAmy) and on EfAmy mutants. Each mutation is indicated by the amino acid in the wild type followed by its position in the primary structure and by the mutated amino acid (data were extrapolated from [37]).

	Relative Activity (%)					Half-Life
α-Amylases	5 °C	25 °C	30 °C	35 °C	60 °C	Hours at 50 °C
Wild type	55%	100%			0%	1.8
E166P	42%	100%			5%	2.4
S185P	45%	100%			0%	2.6
T350P	50%	100%			5%	2.3
V212T	40%	100%			0%	2.0
V232T	40%	100%			5%	2.0
V212T/V232T	25%	100%			5%	2.2
E166P/S185P/T350P	40%		100%		10%	3.0
E166P/S185P/T350P/V212T/V232T	38%			100%	18%	3.3
EcAmy	25%			100%	20%	4.2

**Table 6:** Mean of taxol binding concentrations to infer the relative binding affinities versus β-tubulin isotypes.

β-Tubulin Isotype	Taxol Binding Concentration
*E. crassus* Beta1	0.360 μM
*E. crassus* Beta5	0.765 μM
*E. focardii* Beta1	0.805 μM
*E. focardii* Beta3	0.931 μM
*E. focardii* Beta5	0.220 μM

## Data Availability

Not applicable.

## Supplementary Materials

The following supporting information can be downloaded at https://www.mdpi.com/article/10.3390/md22110497/s1, Figure S1. Compositional trend of each individual amino acid in *E. focardii* (cyan) and *E. crassus* (dark blue) lipases; Table S1. Frequency of individual amino acids and property groups in the lipase sequences from *E. focardii* and *E. crassus*; Table S2. Frequency of individual amino acids in the lipase secondary structures from *E. focardii* and *E. crassus.*

## References

[1] Mangiagalli M., Brocca S., Orlando M., Lotti M. (2020). The “Cold Revolution”. Present and Future Applications of Cold-Active Enzymes and Ice-Binding Proteins. New Biotechnol..

[2] Ramasamy K.P., Mahawar L., Rajasabapathy R., Rajeshwari K., Miceli C., Pucciarelli S. (2023). Comprehensive Insights on Environmental Adaptation Strategies in Antarctic Bacteria and Biotechnological Applications of Cold Adapted Molecules. Front. Microbiol..

[3] Siddiqui K.S., Cavicchioli R. (2006). Cold-Adapted Enzymes. Annu. Rev. Biochem..

[4] Margesin R., Gerday C., Glansdorff N. (2009). Cold-Active Enzymes as New Tools in Biotechnology. Extremophiles.

[5] Hamid B., Bashir Z., Yatoo A.M., Mohiddin F., Majeed N., Bansal M., Poczai P., Almalki W.H., Sayyed R.Z., Shati A.A. (2022). Cold-Active Enzymes and Their Potential Industrial Applications—A Review. Molecules.

[6] Mabrouk S.B., Aghajari N., Ali M.B., Messaoud E.B., Juy M., Haser R., Bejar S. (2011). Enhancement of the Thermostability of the Maltogenic Amylase MAUS149 by Gly312Ala and Lys436Arg Substitutions. Bioresour. Technol..

[7] Watanabe S., Ito M., Kigawa T. (2021). DiRect: Site-Directed Mutagenesis Method for Protein Engineering by Rational Design. Biochem. Biophys. Res. Commun..

[8] Rappaport H.B., Oliverio A.M. (2023). Extreme Environments Offer an Unprecedented Opportunity to Understand Microbial Eukaryotic Ecology, Evolution, and Genome Biology. Nat. Commun..

[9] Rogers A.D. (2007). Evolution and Biodiversity of Antarctic Organisms: A Molecular Perspective. Philos. Trans. R. Soc. B Biol. Sci..

[10] Núñez-Pons L., Avila C., Romano G., Verde C., Giordano D. (2018). UV-Protective Compounds in Marine Organisms from the Southern Ocean. Mar. Drugs.

[11] Pischedda A., Ramasamy K.P., Mangiagalli M., Chiappori F., Milanesi L., Miceli C., Pucciarelli S., Lotti M. (2018). Antarctic Marine Ciliates under Stress: Superoxide Dismutases from the Psychrophilic *Euplotes focardii* Are Cold-Active yet Heat Tolerant Enzymes. Sci. Rep..

[12] Song W., Wilbert N. (2000). Ciliates from Antarctic Sea Ice. Polar Biol..

[13] Agatha S., Wilbert N., Spindler M., Elbrächter M. (1990). Euplotide Ciliates in Sea Ice of the Weddell Sea (Antarctica). Acta Protozool..

[14] Mozzicafreddo M., Pucciarelli S., Swart E.C., Piersanti A., Emmerich C., Migliorelli G., Ballarini P., Miceli C. (2021). The Macronuclear Genome of the Antarctic Psychrophilic Marine Ciliate *Euplotes focardii* Reveals New Insights on Molecular Cold Adaptation. Sci. Rep..

[15] Valbonesi A., Luporini P. (1993). Biology of *Euplotes focardii*, an Antarctic Ciliate. Polar Biol..

[16] Lobanov A.V., Heaphy S.M., Turanov A.A., Gerashchenko M.V., Pucciarelli S., Devaraj R.R., Xie F., Petyuk V.A., Smith R.D., Klobutcher L.A. (2017). Position-Dependent Termination and Widespread Obligatory Frameshifting in Euplotes Translation. Nat. Struct. Mol. Biol..

[17] Pucciarelli S., Sparvoli D., Ballarini P., Piersanti A., Mozzicafreddo M., Arregui L., Miceli C. (2022). Ciliate Microtubule Diversities: Insights from the EFBTU3 Tubulin in the Antarctic Ciliate *Euplotes focardii*. Microorganisms.

[18] Pucciarelli S., Chiappori F., Sparvoli D., Milanesi L., Miceli C., Melki R. (2013). Tubulin Folding: The Special Case of a Beta-Tubulin Isotype from the Antarctic Psychrophilic Ciliate *Euplotes focardii*. Polar Biol..

[19] Pucciarelli S., La Terza A., Ballarini P., Barchetta S., Yu T., Marziale F., Passini V., Methé B., Detrich H.W., Miceli C. (2009). Molecular Cold-Adaptation of Protein Function and Gene Regulation: The Case for Comparative Genomic Analyses in Marine Ciliated Protozoa. Mar. Genom..

[20] La Terza A., Papa G., Miceli C., Luporini P. (2001). Divergence between Two Antarctic Species of the Ciliate Euplotes, *E. focardii* and *E. nobilii*, in the Expression of Heat-Shock Protein 70 Genes. Mol. Ecol..

[21] Kim H., Kim H.-W., Lee J.H., Park J., Lee H., Kim S., Shin S.C. (2022). Gene Family Expansions in Antarctic Winged Midge as a Strategy for Adaptation to Cold Environments. Sci. Rep..

[22] Fojan P., Jonson P.H., Petersen M.T.N., Petersen S.B. (2000). What Distinguishes an Esterase from a Lipase: A Novel Structural Approach. Biochimie.

[23] Joseph B., Ramteke P.W., Thomas G. (2008). Cold Active Microbial Lipases: Some Hot Issues and Recent Developments. Biotechnol. Adv..

[24] Sharma S., Kanwar S.S. (2014). Organic Solvent Tolerant Lipases and Applications. Sci. World J..

[25] Salwoom L., Rahman R.N.Z.R.A., Salleh A.B., Shariff F.M., Convey P., Ali M.S.M. (2019). New Recombinant Cold-Adapted and Organic Solvent Tolerant Lipase from Psychrophilic *Pseudomonas* sp. LSK25, Isolated from Signy Island Antarctica. Int. J. Mol. Sci..

[26] van der Ent F., Lund B.A., Svalberg L., Purg M., Chukwu G., Widersten M., Isaksen G.V., Brandsdal B.O., Åqvist J. (2022). Structure and Mechanism of a Cold-Adapted Bacterial Lipase. Biochemistry.

[27] Yang G., Mozzicafreddo M., Ballarini P., Pucciarelli S., Miceli C. (2021). An In-Silico Comparative Study of Lipases from the Antarctic Psychrophilic Ciliate *Euplotes focardii* and the Mesophilic Congeneric Species Euplotes Crassus: Insight into Molecular Cold-Adaptation. Mar. Drugs.

[28] Zhou H.-X., Pang X. (2018). Electrostatic Interactions in Protein Structure, Folding, Binding, and Condensation. Chem. Rev..

[29] DasSarma S., Capes M.D., Karan R., DasSarma P. (2013). Amino Acid Substitutions in Cold-Adapted Proteins from Halorubrum Lacusprofundi, an Extremely Halophilic Microbe from Antarctica. PLoS ONE.

[30] Yang G., De Santi C., De Pascale D., Pucciarelli S., Pucciarelli S., Miceli C. (2013). Characterization of the First Eukaryotic Cold-Adapted Patatin-like Phospholipase from the Psychrophilic *Euplotes focardii*: Identification of Putative Determinants of Thermal-Adaptation by Comparison with the Homologous Protein from the Mesophilic Euplotes. Biochimie.

[31] Lehmann M., Wyss M. (2001). Engineering Proteins for Thermostability: The Use of Sequence Alignments versus Rational Design and Directed Evolution. Curr. Opin. Biotechnol..

[32] Gupta R., Gigras P., Mohapatra H., Goswami V.K., Chauhan B. (2003). Microbial α-Amylases: A Biotechnological Perspective. Process Biochem..

[33] Pandey A., Nigam P., Soccol C.R., Soccol V.T., Singh D., Mohan R. (2000). Advances in Microbial Amylases. Biotechnol. Appl. Biochem..

[34] Khemakhem B., Ali M.B., Aghajari N., Juy M., Haser R., Bejar S. (2009). Engineering of the α-Amylase from Geobacillus Stearothermophilus US100 for Detergent Incorporation. Biotechnol. Bioeng..

[35] Nielsen J.E., Borchert T.V. (2000). Protein Engineering of Bacterial α-Amylases. Biochim. Biophys. Acta (BBA) Protein Struct. Mol. Enzymol..

[36] van der Maarel M.J., van der Veen B., Uitdehaag J.C., Leemhuis H., Dijkhuizen L. (2002). Properties and Applications of Starch-Converting Enzymes of the α-Amylase Family. J. Biotechnol..

[37] Yang G., Yao H., Mozzicafreddo M., Ballarini P., Pucciarelli S., Miceli C. (2017). Rational Engineering of a Cold-Adapted α-Amylase from the Antarctic Ciliate *Euplotes focardii* for Simultaneous Improvement of Thermostability and Catalytic Activity. Appl. Environ. Microbiol..

[38] Mangiagalli M., Lotti M. (2021). Cold-Active β-Galactosidases: Insight into Cold Adaptation Mechanisms and Biotechnological Exploitation. Mar. Drugs.

[39] Klein W., Weber M.H.W., Marahiel M.A. (1999). Cold Shock Response of *Bacillus Subtilis*: Isoleucine-Dependent Switch in the Fatty Acid Branching Pattern for Membrane Adaptation to Low Temperatures. J. Bacteriol..

[40] Feller G. (2010). Protein Stability and Enzyme Activity at Extreme Biological Temperatures. J. Phys. Condens. Matter.

[41] Chew Y.M., Cross R.A. (2023). Taxol Acts Differently on Different Tubulin Isotypes. Commun. Biol..

[42] Nogales E., Wolf S.G., Downing K.H. (1998). Structure of the Aβ Tubulin Dimer by Electron Crystallography. Nature.

[43] Chiappori F., Pucciarelli S., Merelli I., Ballarini P., Miceli C., Milanesi L. (2012). Structural Thermal Adaptation of β-Tubulins from the Antarctic Psychrophilic Protozoan *Euplotes focardii*. Proteins Struct. Funct. Bioinform..

