# Supplementary material for: Molecular Evolution and Adaptation Strategies in Marine Ciliates: An Inspiration for Cold-Adapted Enzyme Engineering and Drug Binding Analysis
Source: Mar Drugs. 2024 Nov 4;22(11):497. doi: 10.3390/md22110497 (PMC11595582; doi:10.3390/md22110497)
Supplement: Supplementary file 1 [file marinedrugs-22-00497-s001.zip › marinedrugs-3249979-supplementary.pdf]

Supplementary materials

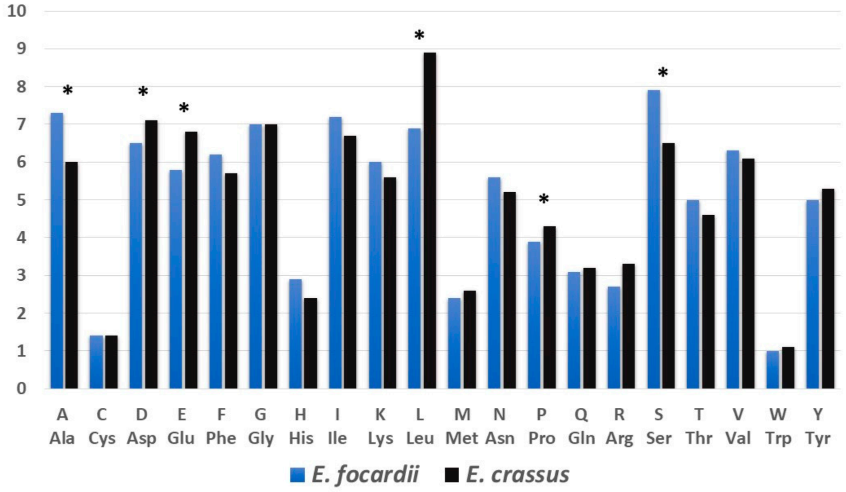

Figure S1. Compositional trend of each individual amino acid in *E. focardii* (cyan) and *E. crassus* (dark blue) lipases. Asterisk indicates significant difference between the two data sets, obtained by Student's t-test. This figure and Figure 1 were obtained from data published by Yang et al. 2021. The lipase genes (104 genes) were extrapolated from the *E. focardii* genome [Nos. MJUV00000000.1] and locally blasted into the *E. crassus* genome (MECR00000000.1) to identify homologous sequences. Lipase sequences were aligned using the T-coffee multiple sequence alignment program. To estimate and compare the amino acid composition of psychrophilic and mesophilic lipases, EMBOSS Pepstats (https://www.ebi.ac.uk/Tools/seqstats/emboss_pepstats/) was used. The amino acids were divided into 12 property groups including, acidic amino acids: Asp and Glu; aliphatic: Ile, Leu, and Val; aromatic: His, Phe, Trp, and Tyr; basic: Arg, His, and Lys; charged: Arg, Asp, Glu, His and Lys; hydrophilic: Asp, Glu, Lys, Asn, Gln, and Arg; hydrophobic: Ala, Cys, Phe, Ile, Leu, Met, Val, Trp, and Tyr; neutral: Gly, Gln, His, Ser, and Thr; non-polar: Ala, Cys, Gly, Ile, Leu, Met, Phe, Pro, Val, Trp, and Tyr; polar: Arg, Asn, Asp, Glu, Gln, His, Lys, Ser, and Thr; small: Ala, Cys, Asp, Gly, Asn, Pro, Ser, Thr, and Val; and tiny: Ala, Cys, Gly, Ser, and Thr. Some of the amino acids are included in more than one property groups. The sum of frequencies of amino acids that fall in each property group was calculated for psychrophilic and mesophilic lipases and compared. The composition data were then analyzed, and a Student's t-test was applied to confirm significant difference between the two data sets (see also Table S1 and S2).

Secondary structural elements in protein sequences were predicted using PSIPRED (http://bioinf.cs.ucl.ac.uk/psipred/). The resulting predictions were used to compute frequencies of different amino acids and property groups of residues in three major secondary structural regions, helix (H), strand (E), and coil (C). The composition data were then analyzed, and a Student's t-test was applied to confirm significant difference between the two data sets.

Table S1. Frequency of individual amino acids and property groups in the lipase sequences from *Euplotes focardii* and *Euplotes crassus* (modified from Yang et al 2021). In the table are reported only the differences that were significant as indicated by t-test p-values. ab: αβ-hydrolase associated lipases; est: Esterase lipases; pat: Patatin-like hospholipases. Avg: Average.

**Amino acids**	***E. focardii***	***E. crassus***	***t*-test**

	ab	est	pat	Avg	ab	est	pat	Avg	*p*-value
Ala	6.7	7.2	8.1	7.3	5.1	5.6	7.3	6.0	**0.037**
Asp	6.3	6.4	7.0	6.5	7.1	6.8	7.4	7.1	**0.048**
Glu	5.4	5.8	6.1	5.8	6.7	6.6	7.2	6.8	**0.016**
Leu	8.0	5.2	7.5	6.9	10.0	7.8	9.0	8.9	**0.023**
Pro	4.4	3.9	3.2	3.9	4.9	4.3	3.6	4.3	**0.001**
Ser	7.5	8.5	7.7	7.9	6.6	6.8	6.0	6.5	**0.029**
**Amino acid group**									
Tiny	27.1	29.6	29.0	28.6	24.6	25.5	26.5	25.5	**0.027**
small	48.9	51.8	51.8	50.8	46.9	48.2	49.5	48.2	**0.036**
acidic	11.7	12.2	13.1	12.3	13.8	13.4	14.6	13.9	**0.022**

Table S2. Frequency of individual amino acids in the lipase secondary structures from *Euplotes focardii* and *Euplotes crassus* (modified from Yang et al., 2021). In the table are reported only the differences that were significant as indicated by t-test p-values. ab: αβ-hydrolase associated lipases; est: Esterase lipases; pat: Patatin-like phospholipase. Avg: Average.

α-helix									
**Amino acids**	***E. focardii***				***E. crassus***				***t*-test *p*-value**
	ab	est	pat	Avg	ab	est	pat	Avg	
Glu	5.3	6.9	7.1	6.4	5.9	7.8	7.9	7.2	**0.014**
Leu	13.2	12.1	10.7	12.0	14.8	13.6	12.9	13.8	**0.014**
β-strand									
Ile	13.6	15.1	15.6	14.8	10.8	12.6	12.5	12.0	**0.004**
Coil									
Ala	5.8	5.5	6.2	5.8	4.3	4.5	5.5	4.8	**0.044**
Asp	9.8	9.1	9.8	9.6	9.2	8.0	8.7	8.6	**0.031**
Gly	9.4	9.6	9.8	9.6	9.1	9.1	9.5	9.2	**0.020**
Pro	6.1	5.3	1.2	4.2	7.0	7.1	3.2	5.8	**0.043**
Ser	9.2	9.7	10.1	9.7	8.3	8.2	8.6	8.4	**0.022**
